# Impact of Multicomponent Training Frequency on Health and Fitness Parameters in Postmenopausal Women: A Comparative Study

**DOI:** 10.3390/healthcare12191980

**Published:** 2024-10-04

**Authors:** Eduardo Martínez-Carbonell, Abraham López-Vivancos, Salvador Romero-Arenas, Fernanda Borges-Silva, Pablo J. Marcos-Pardo, Noelia González-Gálvez, Fco. Javier Orquín-Castrillón

**Affiliations:** 1 IISSEL—Sport and Health Academy, 30562 Murcia, Spain; eduardo.iissel@gmail.com; 2 Facultad de Deporte, Universidad Católica de Murcia (UCAM), 30107 Murcia, Spain; alvivancos@ucam.edu (A.L.-V.); sromero@ucam.edu (S.R.-A.); bsfernanda@ucam.edu (F.B.-S.); ngonzalez@ucam.edu (N.G.-G.); 3 Department of Education, Faculty of Education Sciences, University of Almería, 04120 Almería, Spain; pjmarcos@ual.es

**Keywords:** lipid profile, metabolic health, muscle strength, exercise intervention, balance

## Abstract

Background: Menopause induces physiological changes in women, including increased risks of obesity, cardiovascular diseases, and muscle loss, which can be mitigated by physical exercise. This study aimed to evaluate the effects of a 12-week multicomponent exercise programme, performed 2 or 3 days per week, on health and fitness parameters in postmenopausal women. Methods: Eighty-three postmenopausal women (aged 50–65 years) were randomly assigned to three groups: control group (CG, *n* = 27), 2 days/week exercise group (EG2, *n* = 28), and 3 days/week exercise group (EG3, *n* = 28). The intervention included strength, balance, aerobic, and flexibility exercises. Anthropometric measurements (body weight, BMI, waist-to-hip ratio, lean body mass, body fat percentage), lipid profile, and isometric strength were assessed pre- and post-intervention. Data were analysed using a repeated-measures ANOVA, with *p* < 0.05 considered significant. Results: Significant reductions in body weight, BMI, and waist-to-hip ratio were observed in EG2 and EG3 compared to CG. Lean body mass increased significantly in both EG2 (*p* < 0.001, ES = 1.37) and EG3 (*p* < 0.001, ES = 1.50). EG3 showed a greater reduction in body fat percentage than EG2 (*p* = 0.049). Strength and balance improved significantly in both experimental groups compared to CG, with no significant differences between EG2 and EG3. EG3 also showed superior improvements in lipid profile compared to EG2 and CG. Conclusion: A multicomponent exercise programme, particularly with higher frequency (3 days per week), improves body composition, strength, balance, and lipid profile in postmenopausal women.

## 1. Introduction

The transition to menopause represents a crucial time that profoundly affects women’s physiological and psychological health. Menopause is characterised by a dramatic decline in oestrogen and progesterone levels, leading to numerous adverse effects including the end of reproductive capacity and a wide range of physical and emotional symptoms [1]. This transition leads to significant metabolic changes, such as an increased risk of obesity, alterations in lipid profile, reduced bone density, and an increased incidence of cardiovascular disease [2,3]. In addition, the psychological impact of these hormonal changes can impair women’s quality of life, causing fatigue, anxiety, and depression [4].

One of the most important effects of menopause is a decrease in muscle mass and bone density, which contributes to an increased risk of falls and fractures [4]. These changes are especially pronounced in women who lead a sedentary lifestyle, in whom a lack of physical activity exacerbates muscle and bone deterioration [5,6]. As muscle strength and balance decline, everyday activities become more challenging, compromising mobility and independence.

Physical exercise has been shown to be an effective intervention to mitigate these adverse effects [7]. Different types of physical exercise programmes, particularly those that include strength, cardiovascular, flexibility, and balance training, have shown significant improvements in metabolic health and physical fitness in postmenopausal women [8,9]. Furthermore, the scientific literature suggests that regular exercise can not only improve physical function, but also mental health, alleviating symptoms of anxiety and depression, which are common at this stage of life [10].

Training programmes that combine aerobic and strength training, especially those performed at moderate to high intensities, are considered to be the most effective in improving cardiovascular health and reducing the risk of metabolic diseases [11,12]. These programmes, in addition to improving lipid profile and body composition, also help to preserve or even increase muscle mass, thereby reducing the loss of strength associated with ageing [13]. In this context, multicomponent training programmes, which include several exercise modalities, have gained popularity as a comprehensive strategy to improve both physical and mental health in postmenopausal women [14].

However, there remains a significant gap in the literature regarding the optimal frequency of these multicomponent programmes. Although several studies have demonstrated the benefits of exercising at a frequency of at least twice a week, little is known about whether a higher frequency can result in additional improvements in metabolic health, muscle strength, and balance.

## 2. Materials and Methods

### 2.1. Study Design

This randomised, controlled, parallel-design study evaluated the effects of a multicomponent training programme with two different exercise frequencies (2 vs. 3 days per week) on health and physical fitness in sedentary postmenopausal women. This study was conducted over 12 weeks, with measurements taken at the start and end of the training programme. Participants were randomly assigned to three groups: an experimental group training 2 days per week (EG2), an experimental group training 3 days per week (EG3), and a control group (CG) that did not undertake any intervention. The random assignment was carried out using the Research Randomizer website (https://www.randomizer.org, accessed on 15 January 2024) to ensure an equitable distribution. Participants in the experimental groups completed multicomponent training sessions, which included strength, cardiovascular endurance, flexibility, and balance exercises with moderate to high intensity, individually adjusted through the OMNI-RES scale with Thera-Band^®^ (Theraband, Akron, OH, USA) [15]. The dependent variables evaluated included muscle strength, body composition, lipid profile, and balance. This study was conducted in accordance with the Declaration of Helsinki, and the experimental protocol was approved by the Human Research Ethics Committee of the Catholic University of San Antonio in Murcia, Spain (No. CE031904).

### 2.2. Participants

A total of 83 postmenopausal women (defined as those with >12 months of amenorrhoea) aged 50–65 years were recruited to participate in this study. Participants were randomly assigned to three groups: the CG with 27 subjects, who did not perform any exercise intervention; the EG2 with 28 subjects; and the EG3 with 28 subjects. No formal sample size calculation was initially performed. However, considering the importance of justifying the number of participants, a post hoc power analysis was performed using G*Power software (Version 3.1.9.6, University of Kiel, Germany). Assuming a mean effect size (f = 0.25) for the main study variables, with a significance level α = 0.05 and a total sample size of 83 participants, the statistical power achieved was 0.82. This result indicates that this study had sufficient power to detect significant differences between groups.

The inclusion criteria required the participants to be postmenopausal and lead a sedentary lifestyle, defined as less than 150 min of moderate physical activity per week according to World Health Organization criteria. The exclusion criteria included undergoing drug treatment or taking supplements that could influence the study results, participating in other exercise programmes, not meeting the intervention standards, or attending less than 80% of the sessions during the 12-week study.

### 2.3. Procedure

#### 2.3.1. Body Composition

Body composition was assessed by bioelectrical impedance using the Tanita BC 545N (Hoogoorddreef, Amsterdam, The Netherlands) device, which provided data on fat mass and muscle mass. Waist circumference was measured following the protocols of the International Society for the Advancement of Kinanthropometry (ISAK), using a Cescorf (Porto Alegre, Brazil) metal tape measure with an accuracy of ±1 mm. All measurements were taken before the start of the intervention, during the week prior to the intervention, and were conducted by two ISAK-accredited assessors (Level 1). To minimise possible measurement errors, participants were given specific instructions: to avoid strenuous physical activity during the previous 24 h, to fast for at least 3 h before the assessment, to empty the bladder just before the measurement, and to abstain from alcohol and caffeine consumption in the previous 12 h. Participants remained in the supine position for 10 min prior to bioelectrical impedance measurement, ensuring the correct placement of the electrodes according to the manufacturer’s instructions.

#### 2.3.2. Lipid Profile

Participants’ lipid profile and blood glucose levels were obtained from blood test reports from their referral health centres, both before the start of the intervention programme and at the end of the programme. Analyses included measurement of total cholesterol, high-density lipoprotein (HDL), low-density lipoprotein (LDL), and triglycerides, as well as fasting glucose levels. All blood samples were collected under fasting conditions of at least 12 h, in accordance with international guidelines for the assessment of lipid and glycaemic parameters. Analyses were performed in accredited clinical laboratories following standard and validated procedures, thus ensuring the accuracy and consistency of measurements. Data provided by participants through their medical reports were entered into a database for further statistical analysis.

#### 2.3.3. Maximal Isometric Strength

Maximal isometric strength of the knee extensor and elbow flexor musculature was assessed using a load cell (MuscleLab; Ergotest, Langesund, Norway) connected to the respective measuring devices. The collected data were processed and analysed using MuscleLab 10.190 software. Maximal isometric strength was selected as a reliable and safe measure of muscle strength in sedentary populations, reducing the risk of injury and minimising the influence of movement technique associated with maximal dynamic testing.

For the measurement of maximal isometric strength of the knee extensor musculature, participants were positioned in a chair with the hip and knee at a 90-degree angle. A padded ankle brace, connected to the load cell, was attached to the subject’s ankle. The upper body was supported against the backrest of the chair to ensure postural stability, while the arms remained supported on the side backrests. Participants performed a knee extension without generating momentum, maintaining a maximal contraction for 3 to 5 s. Three attempts were performed, the first being a familiarisation attempt, and a 20 s rest was allowed between attempts. The highest value recorded from the remaining two attempts was selected as the maximal isometric force.

Maximal isometric strength of the elbow flexor musculature was assessed with participants in a standing position, with knees slightly bent and elbows at a 90-degree angle of flexion. A fixed bar connected to the load cell was used, and participants were instructed to hold the bar with both hands and apply maximal isometric elbow flexion force for 3–5 s. As with the knee extension test, three attempts were performed, with the first attempt being a familiarisation attempt, and a 20 s rest between each attempt. The highest value obtained from the final two attempts was recorded as the maximal isometric force.

#### 2.3.4. Balance

Balance was assessed using a force platform connected to a laptop computer equipped with MuscleLab software (Ergotest, Langesund, Norway), designed to analyse participants’ lateral and sagittal oscillations. Following the protocols developed by Onabele et al. [16], participants were instructed to stand on the force platform, focusing their gaze on a fixed point placed at eye level to minimise distraction. Participants were tested in a tandem position, where the dominant leg was placed in front in a heel–toe position. Participants held each position for a maximum of 60 s, with 30 s breaks in a seated position between attempts. Three attempts were made for each position, the first being a familiarisation trial. From the remaining two attempts, the best result was selected for analysis. Data were processed using MuscleLab software.

#### 2.3.5. Experimental Intervention

The training protocol was designed and adapted for women aged 50–65 years for a period of 12 weeks, with a frequency of 2–3 sessions per week, under the supervision of a qualified physical educator. Prior to the start of the main intervention, all participants attended 6 exercise adaptation sessions, with the aim of familiarising them with the protocol and minimising the risk of injury. In addition, each session included an initial activation phase and a specific 10 min warm-up. All physical and neuromotor qualities (i.e., balance, endurance, aerobic capacity, and flexibility) were trained during supervised sessions at a frequency of 2 to 3 times per week.

Control of the training load, including volume, intensity, recovery time, and type of exercise, was carefully monitored, as these are key factors in maximising health benefits and preventing injury. The multicomponent training programme was identical for both intervention groups (EG2 and EG3). Each training session had a total duration of 60–70 min and was structured as follows: (1) balance: 10 min, divided into 2–3 sets of 2–3 exercises, such as the tandem position exercise and centre-of-gravity shifts [14,17]; (2) resistance training: 25 min, divided into 2–3 sets of 15–20 repetitions per exercise, focusing on large muscle groups of both upper and lower body through pushing and pulling movements [14,18,19,20]; (3) endurance training: 20 min, divided into 2–4 sets of 3–5 min per exercise; exercises included global movements such as skateboarding, lateral toe taps, lateral shuffle taps, and moderate- to high-speed walking [11,17,21,22]; (4) flexibility: 10 min, using 4 exercises with 2 repetitions of 30 s per muscle group. The muscle groups involved included the hips, spine, ankles, and shoulders [23,24].

The intensity of the resistance training was programmed at a level of perceived exertion of 8–9 on the OMNI-RES scale, using Thera-Band^®^ elastic bands, where 10 represents maximum intensity [12,15,25]. On the other hand, the intensity of aerobic exercise was determined using the Borg perceived exertion scale (6–20), aiming at a level of 13–15, which corresponds to moderate–high intensity [26]. The aim was to work at a moderate–high intensity, adjusted according to each participant’s subjective perception of exertion.

### 2.4. Statistical Analysis

All data were analysed using SPSS statistical software (version 25.0). The physical characteristics of the subjects are presented as mean ± standard deviation. The normality of the data distribution was verified by the Kolmogorov–Smirnov test, and the homogeneity of variances by Levene’s test.

Training effects were analysed using a two-way ANOVA with repeated measures to compare pre- and post-training scores between groups. Subsequently, selected changes between groups were compared using one-way ANOVA. Effect size (Cohen’s *d*) was calculated and interpreted according to the following criteria: small (<0.3), moderate (0.31–0.5), high (0.51–0.7), very high (0.71–0.9) and almost perfect (>0.9). A *p*-value of *p* < 0.05 was considered statistically significant.

## 3. Results

The results of the pre-test, post-test, and the changes between pre- and post-test for the anthropometric variables assessed are shown in Table 1. Statistically significant differences (*p* < 0.05) were found between the pre- and post-test measurements in both experimental groups for all analysed variables, while the control group showed no significant changes. In the comparison between groups, significant differences were observed in body weight between EG2 and the CG (*p* = 0.028; ES = 0.71), as well as between EG3 and the CG (*p* = 0.003; ES = 1.12). For BMI, significant differences were found between EG2 and CG (*p* = 0.011; ES = 0.79), and between EG3 and CG (*p* = 0.002; ES = 1.19). Lean body mass showed significant differences between EG2 and CG (*p* < 0.001; ES = 1.37), and between EG3 and CG (*p* < 0.001; ES = 1.50). For the waist-to-hip ratio, significant differences were found between EG2 and CG (*p* = 0.004; ES = 1.33), and between EG3 and CG (*p* = 0.024; ES = 0.73). Regarding body fat percentage, significant differences were observed between EG3 and EG2 (*p* = 0.049; ES = 0.65) and between EG3 and CG (*p* < 0.001; ES = 1.37), with no significant differences between EG2 and CG (*p* = 0.070; ES = 0.69). No statistically significant differences were observed between the experimental groups (EG2 and EG3) for the other variables evaluated.

Table 2 presents the results of the pre- and post-test and the variations between the pre- and post-test for lipid profile. Statistically significant differences (*p* < 0.05) were found between the pre- and post-test measurements in both experimental groups for all analysed variables, except for HDL and LDL in EG2. The control group showed no significant changes. In the comparison between groups, significant differences were observed in cholesterol levels between EG2 and the CG (*p* < 0.001; ES = 1.54), as well as between EG3 and the CG (*p* < 0.001; ES = 0.88). No statistically significant differences were observed between the experimental groups (EG2 and EG3) and the control group for the other variables evaluated. Additionally, no significant differences were observed between the experimental groups themselves.

Table 3 shows the results of the pre- and post-test and the variations between pre- and post-test for strength and balance. Statistically significant differences (*p* < 0.05) were found between the pre- and post-test measurements in both experimental groups for all analysed variables, while the control group showed no significant changes. In the comparison between groups, significant differences were observed in isometric leg strength between EG2 and the CG (*p* = 0.004; ES = 0.96), as well as between EG3 and the CG (*p* = 0.001; ES = 1.00). Regarding isometric arm strength, significant differences were found between EG2 and CG (*p* = 0.001; ES = 0.92) and between EG3 and CG (*p* = 0.004; ES = 1.19). Tandem balance showed significant differences between EG2 and CG (*p* < 0.001; ES = 0.42) and between EG3 and CG (*p* < 0.001; ES = 0.52). No statistically significant differences were observed between the experimental groups (EG2 and EG3) and the control group for the other evaluated variables. Additionally, no significant differences were observed between the experimental groups themselves.

## 4. Discussion

The main objective of this study was to evaluate the effects of a 12-week multicomponent training programme with different exercise frequencies (2 vs. 3 days per week) on body composition, lipid profile, muscle strength, and balance in sedentary postmenopausal women. The results showed significant improvements in most of the parameters assessed in both intervention groups (EG2 and EG3), while the control group (CG) showed no relevant changes. Both the 2 days per week group and the 3 days per week group showed significant improvements in most of the parameters assessed, with no significant differences between them, except for fat mass percentage, where EG3 showed a greater reduction compared to EG2. These findings underline the effectiveness of multicomponent training, even at a frequency of 2 days per week, in improving several health markers in postmenopausal women. 

In relation to body composition, both EG2 and EG3 showed significant improvements in body weight, BMI, fat-free mass, and body fat percentage compared to the control group. Although EG3 showed a tendency to improve more than EG2 in terms of these parameters, the differences between the two experimental groups were not significant. These results are consistent with the findings of Colado et al. [12], who also observed similar improvements in body composition in postmenopausal women after a 10-week training programme with different strength modalities using elastic bands and guided machines. In both cases, significant reductions in fat mass and increases in muscle mass were observed, suggesting that different types of strength training may generate similar effects in this population. Additional studies, such as those by Flandez et al. [27], also corroborate that twice-weekly elastic band training can generate significant improvements in total fat mass and fat percentage in postmenopausal women. However, other work, such as that of Kim et al. [28], did not achieve significant results in body composition after 24 weeks of elastic band training in obese postmenopausal women. This disparity in findings could be explained by differences in the duration and intensity of the training programmes.

In our study, both intervention groups (EG2 and EG3) showed significant improvements in most of the parameters evaluated, with no statistically significant differences between them, except in the percentage of fat mass, where EG3 showed a significantly greater reduction than EG2. It is possible that the 12-week intervention period was insufficient to observe additional differences between the different training frequencies. Studies such as that of Magalhães et al. [29] have shown that prolonged (2 years or more) and more frequent (3 days per week) training produces significant reductions in fat mass compared to lower frequency programmes. These findings suggest that, while 2-day-per-week multicomponent training is effective in improving body composition in the short term, higher frequency and programme duration may maximise long-term benefits.

The present study showed that a multicomponent training programme of moderate to high intensity (8–9 on the OMNI-RES scale) with a frequency of two or three sessions per week achieved significant improvements in lipid profile variables. EG2 obtained significant improvements in triglycerides, total cholesterol, and glucose, but did not show relevant changes in HDL-C and LDL-C levels. In contrast, EG3 experienced significant improvements in all lipid profile variables, with highly significant reductions in total cholesterol, HDL-C, LDL-C, and glucose (*p* < 0.001). These results indicate that a moderate- to high-intensity multicomponent training programme is effective in improving the lipid profile of sedentary postmenopausal women, both with a frequency of two and three sessions per week. Although the group that trained three times per week showed significant improvements in all lipid profile variables, the differences between EG2 and EG3 were not statistically significant in most variables, except for total cholesterol. Therefore, both training programmes are effective in improving the lipid profile, and further research is required to determine whether a higher training frequency provides significant additional benefits.

In agreement with our findings, Neves et al. [14] evaluated postmenopausal women after a 16-week programme with a frequency of 3 days per week, obtaining significant improvements in HDL-C (9.5%) but no relevant changes in total cholesterol, LDL-C, or glucose. The difference in the results between that study and the present one could be related to the intensity of the training, as the protocol in this study used a higher intensity, which could have facilitated more robust adaptations in lipid profile. In line with this, Libardi et al. [30] found that 16 weeks of high-intensity strength training generated significant improvements in total cholesterol (21.1%) and LDL-C (69%), but not HDL-C, suggesting that training intensity plays a crucial role in the magnitude of adaptations. On the other hand, not all studies have found significant improvements in lipid profile with exercise. Elliot et al. [31] and Akwa et al. [32] failed to observe changes in lipid profile after resistance and cardiovascular training protocols, respectively, in postmenopausal women. This could be due to variability in the types of exercise, duration of programmes, intensity and adherence of participants. In contrast, Magalhães et al. [29] showed that women who performed strength training for 2 years or more, with a frequency of three sessions per week, experienced significant improvements in HDL-C and total cholesterol. This suggests that training experience and programme duration are also important factors influencing lipid profile adaptations. These findings reinforce the idea that both training intensity and training frequency are key determinants of lipid profile adaptations in postmenopausal women. Programmes with a higher training frequency (3 days per week) and longer duration appear to be more effective in generating significant improvements in lipid profile, highlighting the importance of designing appropriate exercise protocols for this population.

In terms of strength values, both EG2 and EG3 obtained highly significant improvements (*p* < 0.001), with no significant differences between groups. These results suggest that the use of elastic bands is sufficient to induce improvements in maximal strength in postmenopausal women [17,33,34], which is in line with previous studies [18,35]. Although institutions such as the American College of Sports Medicine [36] recommend the use of guided machines for beginner strength training, their accessibility may be limited in some contexts, as in this study. In contrast, elastic bands, such as the Thera-Band^®^, have been shown to be effective in generating neuromuscular adaptations in this population [18,27,35]. In research such as that of Colado et al. [35], the use of elastic bands and guided machines produced similar improvements in muscle strength after 10 weeks of training. These findings reinforce the idea that supervised strength training with elastic bands may be a viable and accessible alternative to improve muscle strength in the first weeks of intervention in postmenopausal women.

In terms of balance, both experimental groups also obtained significant improvements in balance tests. This suggests that a multicomponent training programme, even at a frequency of two sessions per week, is sufficient to improve balance in postmenopausal women. Gillespie et al. [34] support these findings, stating that exercise programmes that include strength, balance, flexibility and cardiovascular endurance training can reduce the risk of falls. Similarly, Otero et al. [37] observed significant improvements in static balance after a 6-month programme combining strength and balance training. In our study, the focus on static balance exercises, such as the tandem stance, may have been key to the observed improvements in balance, particularly in EG3.

It is important to note that, although both groups improved significantly, no significant differences were found between EG2 and EG3 in terms of strength or balance. This suggests that a training frequency of 2 days per week may be sufficient to induce improvements in these parameters in the short term. However, longer-term studies could investigate whether a higher training frequency has additional effects on strength and balance in this population. It would also be interesting to assess the specific exercise volume of standing and tandem exercises to identify the optimal exercise dose to improve balance in postmenopausal women.

## 5. Conclusions

This study demonstrates that a multicomponent training programme, designed specifically for postmenopausal women and performed for 12 weeks, generates significant improvements in body composition, muscle strength, balance, and lipid profile. Both EG2 and EG3 experienced reductions in body weight, BMI, and waist-to-hip ratio, as well as an increase in lean muscle mass and improvements in isometric limb strength. EG3 showed a significantly greater reduction in percent fat mass and total cholesterol levels compared to EG2. This suggests that a higher frequency of training may be more effective in improving certain metabolic parameters in this population. However, no significant differences were observed between EG2 and EG3 in terms of most other variables, indicating that both training frequencies are effective in improving muscle strength, balance, and other markers of health. These findings underscore the importance of regular exercise in managing the physiological changes associated with menopause. In conclusion, a multicomponent training programme, even with a frequency of 2 days per week, is an effective intervention to improve physical and metabolic health in postmenopausal women. A higher frequency of training (i.e., 3 days per week) may enhance benefits in reducing fat mass percentage and lipid profile, specifically total cholesterol. These results may guide the prescription of exercise in postmenopausal women, contributing to an improvement in their quality of life.

## Figures and Tables

**Table 1:** Anthropometric measurements.

	Pre	Post	*p*-Value [95%CI]	Δ	ES
**Weight (kg)**					
**EG2**	68.4 ± 10.88	66.8 ± 11.20	<0.001 [−2.44; −0.76]	−1.6 ± 2.55 ^c^	0.14
**EG3**	67.6 ± 10.13	65.5 ± 9.7	<0.001 [−2.92; −1.24]	−2.8 ± 1.80 ^c^	0.20
**CG**	69.7 ± 9.17	69.7 ± 9.59	1.000 [−0.84; 0.84]	0.0 ± 1.91 ^ab^	0.00
**BMI (kg/m^2^)**					
**EG2**	28.3 ± 4.44	27.5 ± 4.50	<0.001 [−1.07; −0.39]	−0.73 ± 1.05 ^c^	0.16
**EG3**	26.3 ± 3.54	25.4 ± 3.35	<0.001 [−1.21; −0.53]	−0.87 ± 0.70 ^c^	0.24
**CG**	27.6 ± 3.31	27.6 ± 3.56	0.969 [−0.34; 0.33]	−0.01 ± 0.75 ^ab^	0.00
**Lean body mass (kg)**					
**EG2**	39.9 ± 3.95	42.2 ± 4.34	<0.001 [1.65; 3.07]	2.4 ± 1.58 ^c^	0.58
**EG3**	39.3 ± 3.93	42.2 ± 3.74	<0.001 [2.25; 3.67]	3.0 ± 2.09 ^c^	0.73
**CG**	40.6 ± 4.51	40.8 ± 3.92	0.654 [−0.55; 0.87]	0.2 ± 1.62 ^ab^	0.03
**Fat Mass (%)**					
**EG2**	38.3 ± 7.18	36.1 ± 6.64	<0.001 [−3.39; −1.01]	−2.2 ± 3.06 ^b^	0.30
**EG3**	38.4 ± 5.03	34.1 ± 5.53 ^c^	<0.001 [−5.47; −3.09]	−4.3 ± 3.30 ^ac^	0.82
**CG**	38.4 ± 4.56	38.1 ± 4.62 ^b^	0.689 [−1.43; 0.95]	−0.2 ± 2.57 ^b^	0.05
**Waist-to-Hip Ratio (a.u.)**					
**EG2**	0.81 ± 0.06	0.78 ± 0.06 ^c^	0.002 [−0.04; −0.01]	−0.03 ± 0.03 ^c^	0.48
**EG3**	0.82 ± 0.07	0.80 ± 0.06	0.020 [−0.03; −0.00]	−0.02 ± 0.05 ^c^	0.28
**CG**	0.82 ± 0.06	0.83 ± 0.07 ^a^	0.143 [−0.00; 0.03]	0.01 ± 0.03 ^ab^	0.16

^a^: differences with intervention 2 days; ^b^: differences with intervention 3 days; ^c^: differences with control group. BMI: body mass index; CG: control group; EG2: experimental group training two days per week; EG3: experimental group training three days per week; ES: effect size.

**Table 2:** Results of the pre-test, post-test, and the variations between pre- and post-test for lipid profile.

	Pre	Post	*p*-Value [95%CI]	Δ	ES
**Triglycerides (mg/dL)**					
**EG2**	122.0 ± 44.96	111.1 ± 39.31	0.036 [−25.0; 3.18]	−10.9 ± 25.84	0.25
**EG3**	120.4 ± 79.19	103.8 ± 45.97	0.022 [−30.8; −2.58]	−16.7 ± 54.08	0.19
**CG**	127.6 ± 54.83	129.1 ± 63.38	0.964 [−12.7; 15.54]	1.44 ± 12.66	0.01
**Cholesterol (mg/dL)**					
**EG2**	214.7 ± 42.47	194.8 ± 30.07	<0.001 [−29.2; −10.63]	−19.9 ± 22.41 ^c^	0.45
**EG3**	222.6 ± 39.04	202.3 ± 22.70	<0.001 [−29.6; −10.93]	−20.24 ± 32.94 ^c^	0.50
**CG**	209.5 ± 41.52	212.3 ± 40.92	0.551 [−6.5; 12.11]	2.8 ± 6.90 ^ab^	0.07
**HDL (mg/dL)**					
**EG2**	63.6 ± 14.96 ^b^	67.1 ± 14.14	0.068 [−0.3; 7.17]	3.4 ± 11.58	0.22
**EG3**	53.5 ± 12.4 ^ac^	63.0 ± 12.56	<0.001 [5.8; 13.25]	9.5 ± 10.94	0.74
**CG**	63.0 ± 11.94 ^b^	63.4 ± 10.89	0.778 [−3.3; 4.13]	0.4 ± 2.99	0.03
**LDL (mg/dL)**					
**EG2**	133.4 ± 36.42	125.36 ± 32.35	0.800 [−17.1; 0.98]	−8.0 ± 24.39	0.42
**EG3**	137.6 ± 34.26	120.68 ± 21.67	<0.001 [−25.9; −7.91]	−16.9 ± 28.51	0.16
**CG**	129.8 ± 31.09	131.68 ± 26.91	0.685 [−7.2; 10.86]	1.8 ± 11.22	0.00
**Glucose (mg/dL)**					
**EG2**	88.1 ± 6.49	85.3 ± 6.54	0.018 [−5.28; −0.23]	−2.8 ± 4.45	0.46
**EG3**	93.5 ± 12.14	87.2 ± 10.10	<0.001 [−8.77; −3.71]	−6.2 ± 9.26	0.53
**CG**	87.1 ± 9.18	88.4 ± 11.49	0.371 [−1.17; 3.89]	1.4 ± 3.89	0.11

^a^: differences with two-day intervention; ^b^: differences with three-day intervention; ^c^: differences with control group. CG: control group; EG2: experimental group training two days per week; EG3: experimental group training three days per week; ES: effect size; HDL: high-density lipoprotein; LDL: low-density lipoprotein.

**Table 3:** Results of the pre- and post-test and the variations between pre- and post-test for strength and balance.

	Pre	Post	*p*-Value [95%CI]	Δ	ES
**Isometric Strength with Knee at 90° (N)**					
**EG2**	230.2 ± 93.51	297.5 ± 113.03	0.001 [36.54; 98.11]	67.3 ± 88.8 ^c^	0.70
**EG3**	280.0 ± 115.69	345.0 ± 0.87 ^c^	0.001 [34.20; 95.77]	65.0 ± 80.2 ^c^	0.54
**CG**	281.4 ± 99.24	275.7 ± 84.15 ^b^	0.714 [−36.46; 25.11]	−5.7 ± 59.67 ^ab^	0.06
**Isometric Strength with Elbow at 90° (N)**					
**EG2**	143.0 ± 56.95	200.2 ± 67.30	0.001 [35.42; 78.98]	57.2 ± 72.7 ^c^	0.97
**EG3**	180.0 ± 33.86	230.2 ± 43.52 ^c^	0.001 [28.38; 71.93]	50.2 ± 30.4 ^c^	1.43
**CG**	180.0 ± 72.83	179.0 ± 60.21 ^b^	0.930 [−22.74; 20.81]	−1.0 ± 52.4 ^ab^	0.01
**Tandem Balance (s)**					
**EG2**	6.8 ± 1.76	8.1 ± 2.86	0.005 [6.76; 9.39]	1.3 ± 2.21 ^c^	0.63
**EG3**	7.1 ± 1.33	9.5 ± 3.88	0.001 [8.16; 10.80]	2.4 ± 2.65 ^c^	0.83
**CG**	8.4 ± 3.10	9.0 ± 2.58	0.192 [−7.07; 9.71]	0.6 ± 3.67 ^ab^	0.21

^a^: differences with two-day intervention; ^b^: differences with three-day intervention; ^c^: differences with control group. CG: control group; EG2: experimental group training two days per week; EG3: experimental group training three days per week; ES: effect size.

## Data Availability

The datasets generated and analysed during the current study are not publicly available but are available from the corresponding author who organised this study.
